# Suppression of PKCδ/NF-κB Signaling and Apoptosis Induction through Extrinsic/Intrinsic Pathways Are Associated with Magnolol-Inhibited Tumor Progression in Colorectal Cancer In Vitro and In Vivo

**DOI:** 10.3390/ijms21103527

**Published:** 2020-05-16

**Authors:** Chun-Min Su, Yueh-Shan Weng, Lin-Yen Kuan, Jiann-Hwa Chen, Fei-Ting Hsu

**Affiliations:** 1 Department of Surgery, Show Chwan Memorial Hospital, Changhua 500, Taiwan; soajimmy@gmail.com; 2 Department of Biological Science and Technology, China Medical University, Taichung 404, Taiwan; joyceweng@nhri.gov.tw; 3 Department of Emergency Medicine, Cathay General Hospital, Taipei 106, Taiwan; linyenrr@yahoo.com.tw (L.-Y.K.); cgh08335@cgh.org.tw (J.-H.C.); 4 School of Medicine, Fu Jen Catholic University, New Taipei City 242, Taiwan

**Keywords:** PKCδ, NF-κB, magnolol, apoptosis, colorectal cancer

## Abstract

Magnolol is one of the hydroxylated biphenyl compounds from the root and stem bark of *Magnolia officinalis*, which shown to possess anti-colorectal cancer (CRC) effects. However, the regulatory mechanism of magnolol on apoptosis and NF-κB signaling in human CRC has not been elucidated. Thus, we investigated the inhibitory mechanism of magnolol on human and mouse CRC (HT-29 and CT-26) in vitro and in vivo. Results from reporter gene assay indicated that both magnolol and rottlerin (PKCδ inhibitor) reduced the endogenous NF-κB activity. In addition, indolactam V (PKCδ activator)-induced NF-κB signaling was significantly suppressed with both magnolol and rottlerin treatment. Results from Western blotting also indicated that phosphorylation of PKCδ and NF-κB -related proteins involved in tumor progression were effectively decreased by magnolol treatment. The invasion capacity of CRC cells was also attenuated by both magnolol and rottlerin. Furthermore, magnolol triggered Fas/Fas-L mediated extrinsic apoptosis and mitochondria mediated intrinsic apoptosis were validated by flow cytometry. Most importantly, tumor growth in both HT-29 and CT-26 bearing mice were suppressed by magnolol, but no pathologic change was detected in mice kidney, spleen, and liver. As confirmed by immunohistochemistry (IHC) staining from tumor tissue, PKCδ/NF-κB signaling and downstream proteins expression were decreased, while apoptotic proteins expression was increased in the magnolol treated group. According to these results, we suggest that the induction of apoptosis through extrinsic/intrinsic pathways and the blockage of PKCδ/NF-κB signaling are associated with the magnolol-inhibited progression of CRC.

## 1. Introduction

Colorectal cancer (CRC) is a commonly diagnosed cancer and the fourth leading cause of cancer death in the world [1]. Epidemiological studies indicated obesity, a lack of dietary fiber intake, low physical activity, and smoking as unfavorable risk factors are associated with the formation of colorectal cancer [2,3]. For the improvement of survival outcomes of patients with CRC, more effective adjuvant therapy has been developed. New treatment approaches, including targeted therapy and immunotherapy, have been shown to prolong overall survival in CRC patients and approved for treatment of CRC [1,4,5].

Chinese herbal medicines (CHMs), including natural compounds and composite formulae derived from a single herb or combination of herbs, have been recognized as complementary adjuvant therapy to diminish chemotherapy-induced side effects and significantly increase overall survival in patients with CRC in China [6,7]. A high intake of natural compounds such as flavonoids, lignans, and isoflavones was correlated with the reduction of CRC risk [8,9]. Anti-CRC mechanism of effective compounds and composite formulas has been investigated by using cells and animal models [10,11].

Nuclear factor kappa-light-chain-enhancer of activated B cells (NF-κB), the oncogenic transcription factor composed of p50 and p65 subunits, governs various hallmarks of cancer though regulating transcription of NF-κB target genes. Active NF-κB signaling potentiates the expression of proliferation, survival, angiogenesis, and metastasis-related proteins encoded by NF-κB target genes leading to development and progression of CRC [12,13]. Some studies revealed NF-κB signaling can be activated by upstream kinases involved in tumor progression such as protein kinase B (PKB/AKT), mitogen-activated protein kinase (MAPKs), or protein kinase C delta (PKCδ) in CRC [14,15,16].

Apoptosis, the form of programmed cell death, is elicited with extrinsic and intrinsic stimuli through caspases-mediated extrinsic and intrinsic death pathway [17]. Both the blockage of NF-κB signaling and induction of apoptosis have been indicated to participate in the inhibition of CRC progression by CHMs [10,18]. *Coptidis rhizome*, the traditional herb used for treatment of gastrointestinal disease, halted growth of CRC by apoptosis through intrinsic apoptotic pathway [19]. Curcumin, the multifunctional flavonoid isolated from *Curcuma longa*, was reported to disrupt cell cycle progression through inhibition of NF-κB pathway in CRC [20].

Use of *Magnolia officinalis* Rehd. Et Wils, the medicinal herb, has been suggested as associated with the obvious improvement of survival in patients with metastatic CRC. Both honokiol and magnolol, lignans, are major bioactive ingredients of *Magnolia officinalis Rehd Et Wils* [21]. Honokiol has been shown to induce apoptosis through extrinsic/intrinsic pathways and inhibit NF-κB activity in CRC [22,23]. Dephosphorylation of ERK is essential for magnolol-attenuated NF-κB activity in hepatocellular carcinoma cells [24]. In Hsu’s study, magnolol was shown to increase the phosphorylation of ERK in CRC COLO205 cells as a short term effect (within 60 min) [25]. However, the regulatory mechanism of magnolol on apoptosis and the long-term effect on NF-κB signaling in CRC remains unclear. The major purpose of present study was to investigate whether magnolol induces apoptosis through extrinsic/intrinsic pathways and downregulate PKCδ/NF-κB signaling in CRC in vitro and in vivo.

## 2. Results

### 2.1. Both Magnolol and PKC Inhibitor May Suppress NF-κB Signaling in CRC Cells

**Figure 1:**
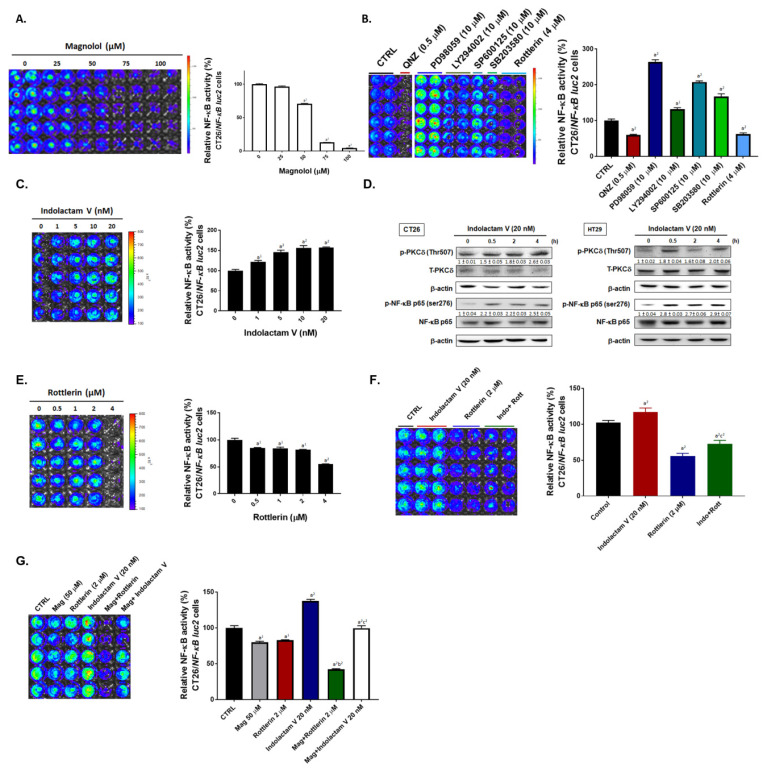
The activation of NF-κB is suppressed by magnolol through inhibition of PKCδ signaling transduction in CRC cells. (**A**) NF-κB reporter gene assay result after 0–100 μM magnolol treatment is displayed by luminesce image and quantification bar chart. (a^1^ *p* < 0.05 and a^2^ *p* < 0.01 vs. 0 μM magnolol) (**B**) NF-κB luminesce image and quantification bar chart after treated with 0.5 μM QNZ (NF-κB inhibitor), 10 μM PD98059 (ERK inhibitor), 10 μM LY294002 (AKT inhibitor), 10 μM SP600125 (JNK inhibitor), 10 μM SB203580 (p38 inhibitor) and 4 μM Rottlerin (PKCδ inhibitor) is shown. (a^1^ *p* < 0.05 and a^2^ *p* < 0.01 vs. non-treated control) (**C**,**D**) NF-κB luminesce image, quantification bar chart and Western blotting results after treated with 0–20 nM Indolactam V (PKC activator). (a^1^ *p* < 0.05 and a^2^ *p* < 0.01 vs. non-treated control) (**E**–**G**) NF-κB luminesce image and quantification bar chart after or magnolol 50 μM, 0–4 μM Rottlerin, 20 nM Indolactam V or combined treatment. (a^1^ *p* < 0.05 and a^2^ *p* < 0.01 vs. non-treated control; b^2^ *p* < 0.01 vs. Rottlerin single treatment; c^2^ *p* < 0.01 vs. Indolactam V single treatment).

We investigated the effect and inhibitory mechanism of magnolol on NF-κB activity in CRC. First, NF-κB activation of CT-26 cells was evaluated by using an NF-κB reporter gene assay 24 h after treatment with different concentrations of magnolol, NF-κB inhibitor (QNZ), or different types of kinase inhibitor (ERK inhibitor (PD98059), AKT inhibitor (LY294002), JNK inhibitor (SP600125), P38 inhibitor (SB203580), PKCδ inhibitor (Rottlerin). As illustrated in NF-κB reporter gene assay results, magnolol may suppress NF-κB activity as dose-dependent manner (Figure 1A). Next, we evaluated the effect of PKC activator (indolactam V) on NF-κB signaling and the phosphorylation of PKCδ. Indolactam V not only significantly induced NF-κB signaling, but also augmented the phosphorylation of PKCδ in a dose dependent manner (Figure 1C,D). In addition, we found that Indolactam V induced NF-κB activity may be diminished by PKC inhibitor (Rottlerin) (Figure 1F). Finally, we verified whether magnolol attenuated indolactam V-induced NF-κB signaling. Importantly, we found that indolactam V-induced NF-κB signaling was effectively inhibited by magnolol treatment (Figure 1G). In sum, NF-κB signaling was decreased by both magnolol and PKCδ inhibitor.

### 2.2. Magnolol Suppressed Tumor Cell Growth, PKC/NF-κB Signaling, Expression of NF-κB Mediated Downstream Proteins in CRC Cells

**Figure 2:**
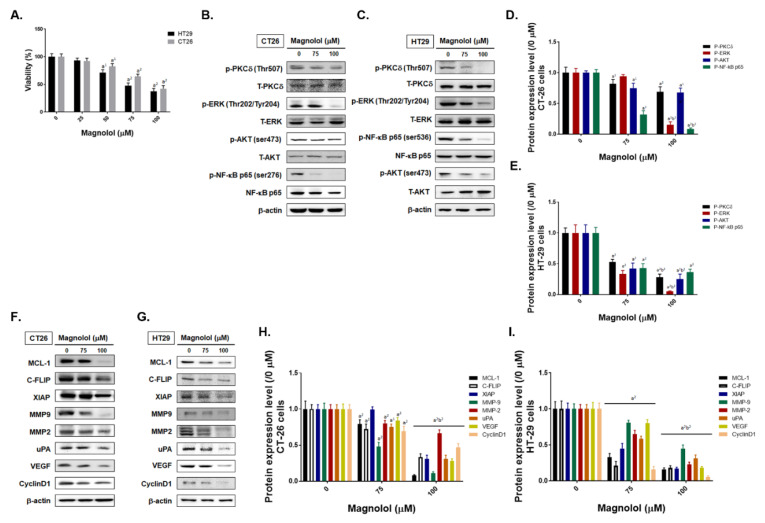
The viability, the phosphorylation of PKCδ/ERK/AKT/NF-κB and the expression of NF-κB mediated downstream proteins is suppressed by magnolol in CRC cells. (**A**) MTT assay result of magnolol is presented. Western blotting and three repeated PKCδ, ERK, AKT, NF-κB protein expression average level bar chart in (**B**) CT26 and (**C**) HT29 after magnolol treatment are displayed. (**D**,**E**,**H**,**I**) Repeated experiment of protein expression level is calculated and presented. Western blotting results of NF-κB mediated downstream proteins on (**F**) CT26 and (**G**) HT29 after magnolol treatment is shown. (a^2^ *p* < 0.01 vs. 0 μM magnolol; b^2^ *p* < 0.01 vs. 75 μM magnolol).

In Figure 2A, we identified the toxicity effect of magnolol in CT26 and HT29 cells. The IC_50_ of magnolol in CT26 and HT29 cells was around 75 μM at 24 h. Next, we identified whether the phosphorylation of PKCδ, ERK, AKT, and NF-κB was altered by magnolol in CRC cells. In both CT26 and HT29 CRC cells, magnolol can effectively dephosphorylate PKCδ, ERK, AKT and NF-κB molecules (Figure 2B,C). Western blotting quantification results also illustrated the phosphorylation of these molecules was markedly decreased by magnolol by dose depend manner (Figure 2D,E). Furthermore, we identified the alteration of NF-κB downstream proteins expression after magnolol treatment. As showed in Figure 2F–I, expression of NF-κB downstream proteins including MCL-1, C-FLIP, XIAP, MMP-2, MMP-9, VEGF, uPA, and CyclinD1 were all significantly reduced by magnolol [26,27,28,29]. Taken together, magnolol induced the inhibition of CRC cells proliferation, the suppression of PKC-δ/NF-κB signaling, and decreasing of NF-κB downstream protein expression.

### 2.3. Magnolol Triggered Both Extrinsic and Intrinsic Apoptosis Effect in CRC Cells

**Figure 3:**
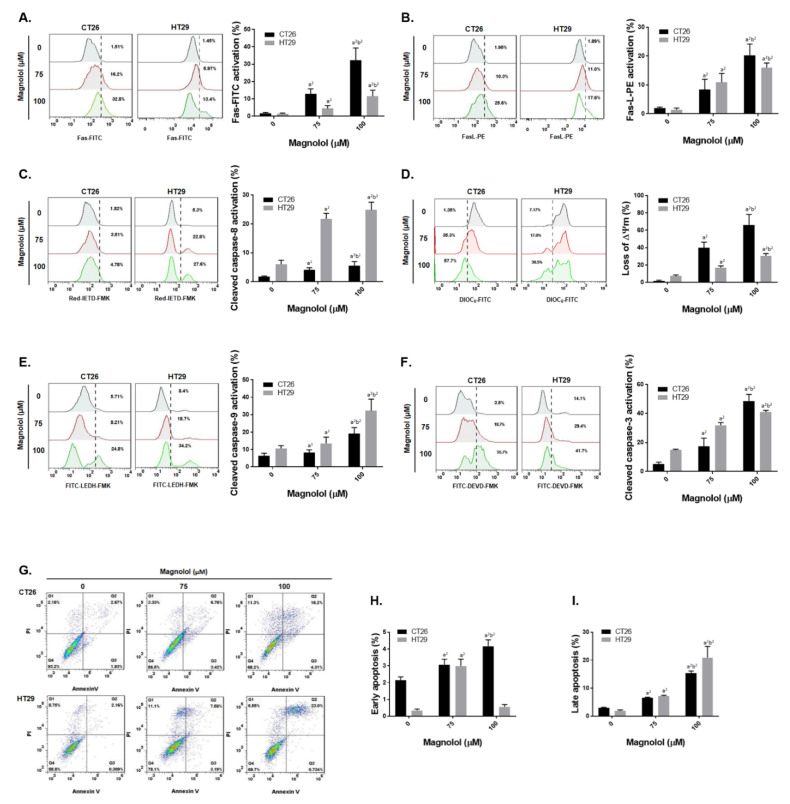
Both death receptor and mitochondria dependent apoptosis effect is induced by magnolol in crc cells. The activation status of apoptosis markers including (**A**) Fas, (**B**) Fas-L, (**C**) cleaved caspase-8, (**D**) loss of ΔΨm, (**E**) cleaved caspase-9 (**F**) cleaved caspase-3 (**G**–**I**) Annexin-V/PI has shown by one represented histogram pattern from each group and average repeated experiment chart. All experiments were performed in triplicate; each was repeated at least three times (a^1^ *p* < 0.05 and a^2^ *p* < 0.01 vs. 0 μM magnolol; b^1^ *p* < 0.05 and b^2^ *p* < 0.01 vs. 75 μM magnolol).

We further evaluated the regulatory mechanism of magnolol on apoptosis in CRC cells. Fas/Fas-L, the death receptor and its ligand, mediated apoptosis were both activated by magnolol treatment in CT26 and HT29 cells (Figure 3A,B). In Figure 3C, cleaved caspase-8 was increased by magnolol. Moreover, magnolol also increased the loss of ΔΨm and the activated caspase-9 (Figure 3D,E). Subsequently, caspase-3 was activated 20–40% by the magnolol treatment group. Annexin-V and PI double positive population, defined as late apoptosis and necroptosis (Figure 3G,I), was also effectively induced by magnolol. In the context of the above results, we suggested that magnolol may trigger both death receptor and mitochondria dependent apoptosis mechanism in CRC cells.

### 2.4. Inhibition of PKCδ/NF-κB Signaling Was Associated with Magnolol-Diminished Invasion Ability of CRC Cells

**Figure 4:**
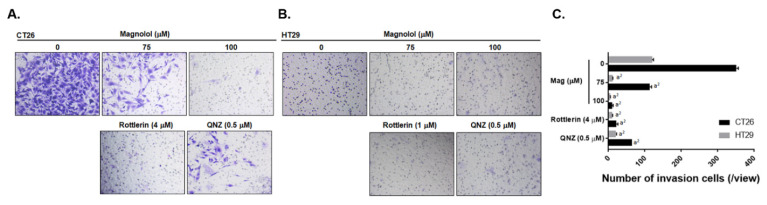
Tumor invasion capacity is suppressed by magnolol in CRC cells. One represented transwell invasion membrane from each group and average of repeated result in (**A**,**B**) CT26 and (**C**) HT29 is displayed. (a^2^ *p* < 0.01 vs. 0 μM magnolol).

In addition to apoptosis effect, we further investigated whether inhibition of PKC-δ/NF-κB signaling was implicated with magnolol-abolished invasion ability of CRC cells. The transwell invasion assay was performed in CT26 and HT29 cells after magnolol, Rottlerin, and QNZ treatment. As showed in Figure 4A,C, the number of invasion CT26 cells was reduced by magnolol in a dose dependent manner. Furthermore, after Rottlerin and QNZ treatment the invasion CT26 cells percentage was also significantly decreased. In the meantime, HT29 also showed a similar invasion reduction effect after magnolol, Rottlerin, and QNZ treatments (Figure 4B,C). These results were corresponded to the reduction of invasion related proteins expression after magnolol treatment in Figure 2F,I. In conclusion, the inhibition of PKC-δ/NF-κB signaling may participate in magnolol-disrupted the invasion capacity of CRC.

### 2.5. Magnolol Effectively Suppressed CRC-Bearing Tumor Growth

**Figure 5:**
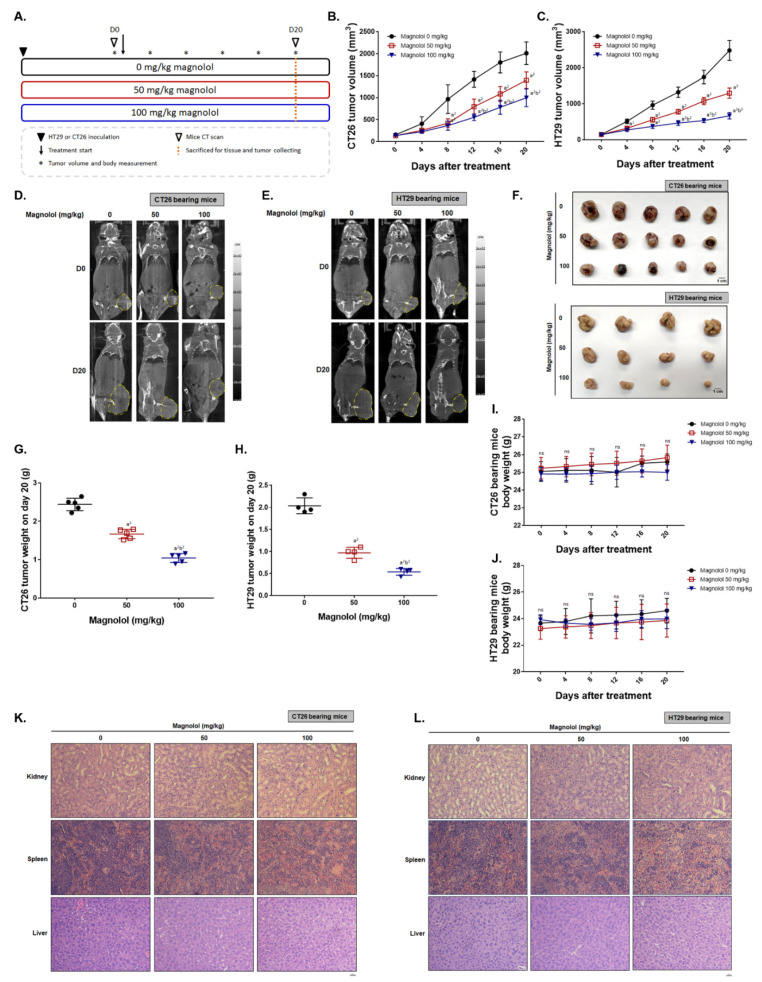
CRC growth is suppressed by magnolol treatment as dose-dependent manner. (**A**) Experimental flow chart of CT26 and HT29 bearing mice. Treatment is started after 3 weeks of tumor inoculation, and tumor volume reached average 100 mm^3^. (**B**,**C**) Tumor volume, (**D**,**E**) CT scanning images, (**F**) isolated tumor images, (**G**,**H**) isolated tumor weights, and (**I**,**J**) body weights in CT26 and HT29 bearing mice. (**K**,**L**) One represented pathology image of kidney, spleen and liver from each group. (a^2^ *p* < 0.01 vs. 0 mg/kg magnolol; b^2^ *p* < 0.01 vs. 50 mg/kg magnolol).

To confirm the anti-tumor effect of magnolol, we established both CT26 and HT29 bearing animal models. As illustrated in Figure 5A, HT29 or CT26 cells were inoculated into mice right flank and treated with various dose of magnolol for 20 days. The detailed animal experiment procedure is displayed in Figure 5A accompanied by a subtitled description. The tumor volume of CT26 and HT29 was markedly decreased by magnolol (Figure 5B,C). After eight days of treatment, a significant difference between non-treated and magnolol treated groups was found. A CT scan was performed to confirm tumor growth after treatment. Figure 5D,E shows that the CT scanning results from CT26 and HT29 bearing mice all indicated the inhibition of tumor growth on magnolol treatment. With a higher dose of magnolol, a tumor isolated from CT26 and HT29 bearing mice on day 20 revealed the marked shrinkage as compared to other groups (Figure 5F). Furthermore, the isolated tumor from each group was measured by scale. Results in Figure 5G–H indicated the smallest tumor weight was found in higher dose of magnolol. Then, we further identified whether magnolol treatment may cause the general toxicity or the pathology change on mice kidney, spleen and liver tissue. Body weight recorded from day 0–20 in CT26 and HT29 bearing mice didn’t show any noticeable change (Figure 5I,J). Pathology H&E staining results also didn’t indicate any alteration between non-treated and magnolol treated mice (Figure 5K,L). These results suggested that magnolol may suppress tumor growth without inducing general toxicity in mice.

### 2.6. Magnolol Suppressed PKCδ/NF-κB Signaling, NF-κB Related Downstream Proteins Expression and Promoted Apoptotic Aroteins Axpression

**Figure 6:**
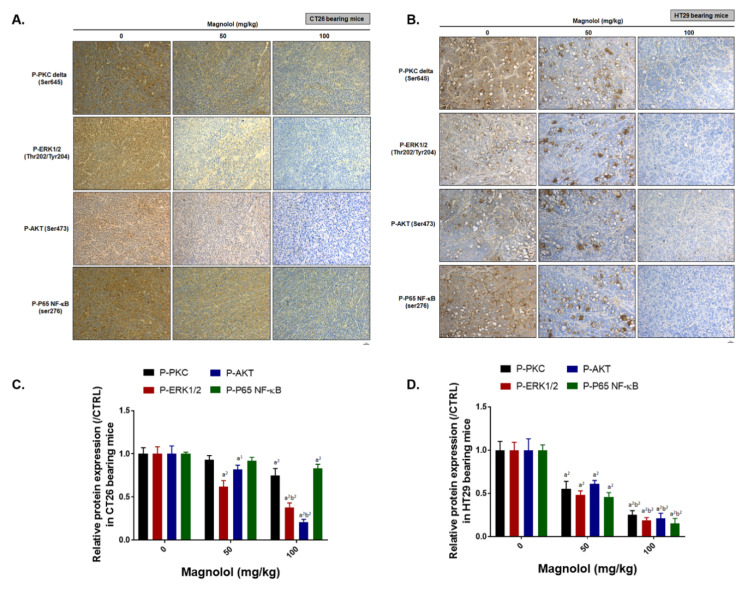

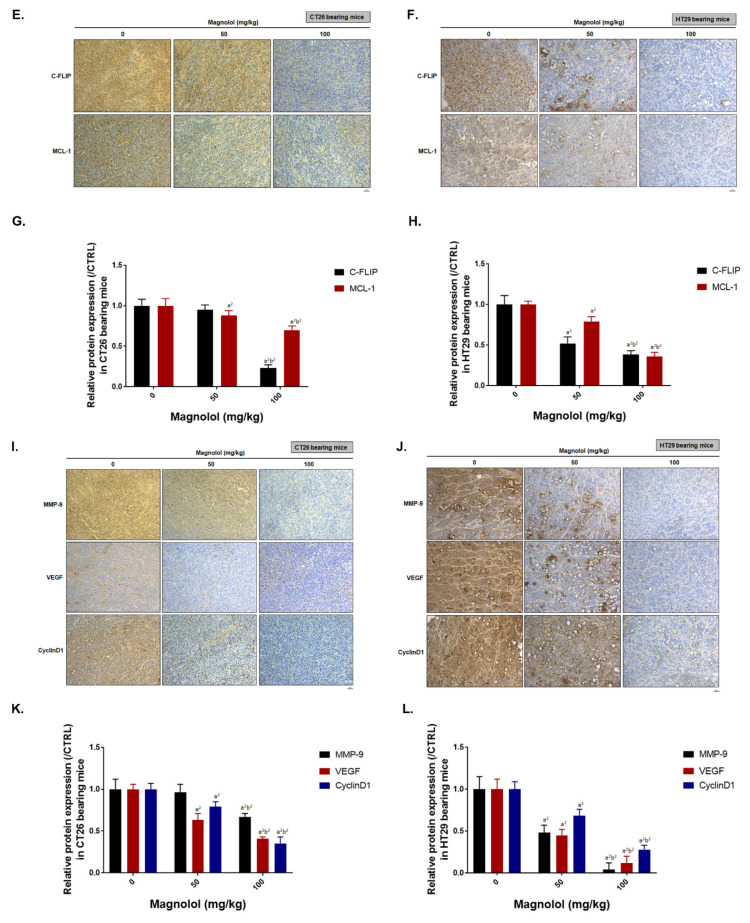

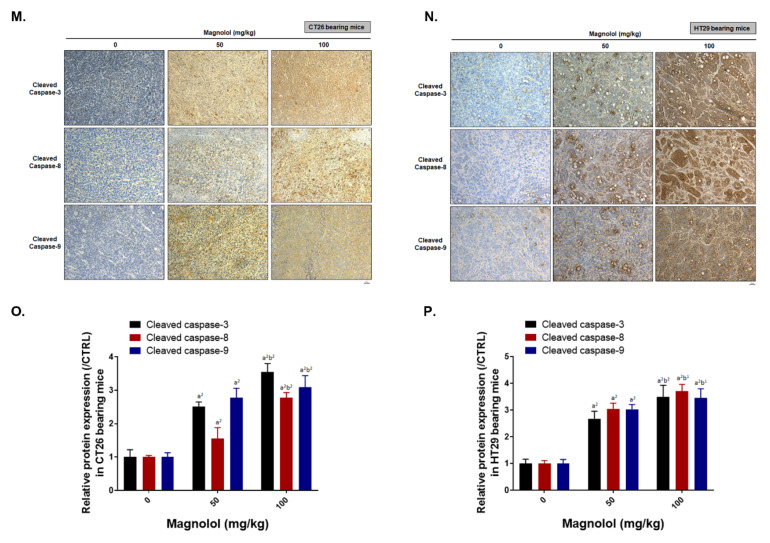
CRC growth is suppressed by magnolol via inhibition of PKCδ/NF-κB pathway. (**A**–**D**) One represented IHC image and protein phosphorylation levels of PKCδ, ERK, AKT, and NF-κB in CT26 and HT29 tumor. (**E**–**H**) One represented IHC image and proteins expression quantification results of C-FLIP and MCL-1 in CT26 and HT29 tumor. (**I**–**L**) One represented IHC image and proteins expression quantification results of MMP-9, VEGF and CyclinD1 proteins in CT26 and HT29 tumor. (**M**–**P**) One represented IHC image and proteins expression quantification results of cleaved-caspase-3, -8 and -9 in CT26 and HT29 tumor. Three tumor tissues are performed with IHC staining from each group and repeated twice. (a^1^, a^2^ *p* < 0.01 vs. 0 mg/kg magnolol; b^2^ *p* < 0.01 vs. 50 mg/kg magnolol).

Finally, we investigated the alteration in expression of PKCδ/NF-κB, NF-κB regulated downstream proteins and apoptotic proteins in CRC tissues by IHC staining after magnolol treatment. The phosphorylation of PKCδ, ERK, AKT, and NF-κB was decreased by magnolol in CT26 and HT29 bearing mice (Figure 6A–D). In Figure 6E–H, the anti-apoptosis related proteins that regulated by NF-κB such as C-FLIP and MCL-1 were also diminished by magnolol. Additionally, invasion related and angiogenesis related proteins that regulated by NF-κB, such as MMP-9 and VEGF, were also effectively suppressed by magnolol (Figure 6I–L). Additionally, magnolol may reduce the protein expression of cyclinD1, the proliferation related molecule. In sum, our results elucidated that magnolol may not only suppress PKCδ/NF-κB mediated tumor progression but trigger extrinsic/intrinsic apoptosis related proteins expression (Figure 6M–P). Death receptor dependent molecules such as Fas, Fas-L, and cleaved caspase-8 were all increased by magnolol. Moreover, the loss of ΔΨm and the activated caspase-9, both recognized as mitochondria-dependent apoptosis processes or markers, was also increased by magnolol. Cleaved-caspase-3, an apoptosis marker, was also markedly induced by magnolol treatment. In sum, magnolol not only constrained PKCδ/NF-κB signaling, but also triggered apoptotic (cleaved-caspase-3, -8, and -9) proteins expression in CRC tissues.

## 3. Discussion

Protein kinase C (PKC), the family of serine/threonine kinases, regulates oncogenic signaling transduction relevant to tumor cell proliferation, survival, and invasion [30,31]. PKC-delta (PKCδ), the PKC isozyme, has been demonstrated to be an upstream modulator of NF-κB which can promote the expression of NF-κB-mediated anti-apoptotic oncogenes cIAP-2 and C-FLIP in colorectal cancer cells [16,31]. Our results showed that PKCδ inhibitor significantly reduced NF-κB activity, whereas AKT or MAPKs inhibitor did not (Figure 1B). We also found magnolol suppressed PKC activator-induced NF-κB signaling and phosphorylation of PKCδ in CRC cells (Figure 1C,D). Notably, the phosphorylation of PKCδ was markedly decreased by magnolol treatment in both CT-26 and HT-29 cells (Figure 2B–E). Those results indicated that PKCδ inactivation is required for magnolol-inhibited NF-κB signaling in CRC. In addition to tumor progression, constitutive activation of NF-κB also mediates resistance to chemotherapy or radiotherapy in CRC. Inhibition of NF-κB signaling has been shown to potentiate anti-CRC efficacy of 5-Fluorouracil (5-FU), the chemotherapeutic agent, or radiation [32,33,34]. We suggest the combination of magnolol and 5-Fu or radiation as a novel potential strategy which may offer therapeutic benefits for patients with CRC.

AKT or ERK is the critical component of phosphoinositide 3-kinase (PI3K)/AKT or RAF/mitogen-activated protein/extracellular signal-regulated kinase (MEK)/ERK signaling pathway, respectively. The high expression of both ERK and AKT phosphorylation was required for the progression of CRC and were correlated with a poor prognosis in patients with CRC [35,36,37]. Hsu et al. presented magnolol induced p21 expression and cell cycle arrest through upregulation of ERK activation in colon cancer COLO-205 cells (within 60 min) [25]. Thus, the increased phosphorylation of ERK may be involved in magnolol-induced early response in CRC. In our data presented the protein levels of both ERK and AKT phosphorylation were significantly reduced by magnolol treatment in both CT-26 and HT-29 cells (24 h) and tumor tissue (Figure 2B–E and Figure 6A–D). Hua et al. also reported that Honokiol may reduce the phosphorylation of AKT, ERK, and NF-κB p65 in HT-29 cells [23]. Our results also demonstrate that magnolol decreased the phosphorylation of AKT, ERK, and NF-κB p65 in HT-29 cells (Figure 2C).

The overexpression of oncogenic proteins, such as Cyclin-D1, MCL-1, C-FLIP, XIAP, MMP-9, MMP-2, uPA, and VEGF, are correlated with constitutive NF-κB activity and involved in promoting tumor progression in CRC [26,27,28,29]. Cyclin-D1 is the cell cycle protein which mediates cell cycle transition from G_1_ to S phase [27]. MMP-9, MMP-2, uPA, and VEGF, as metastasis and angiogenesis-associated proteins, promote extracellular matrix degradation and new blood vessel development, leading to enhanced tumor invasion and growth [26,27,28,29]. Protein levels of Cyclin-D1, MCL-1, C-FLIP, XIAP, MMP-9, MMP-2, uPA, and VEGF were suppressed with magnolol treatment (Figure 2F–I and Figure 6E–L). Magnolol significantly inhibited tumor growth and invasion in CRC. In addition, the invasion ability of CRC was also abolished by both PKCδ and NF-κB inhibitors (Figure 4).

The activity or expression of caspase-3, caspase-9, or FAS was frequently reduced and associated with a poor outcome in CRC patients [38,39,40]. Extrinsic and intrinsic pathway-initiated apoptosis can be halted by the decreased expression of apoptotic proteins and increased expression of anti-apoptotic proteins [41]. MCL-1, XIAP, and C-FLIP are anti-apoptotic proteins which mediate acquired resistance to therapeutic agents or radiotherapy in CRC [42,43,44]. Lin et al. presented magnolol induced apoptosis through extrinsic and intrinsic pathways and inhibited B-cell lymphoma 2 (BCL-2) expression in HCC Hep-G2 cells [45]. According to our results, apoptosis and extrinsic/intrinsic apoptotic signaling transduction (the activation of Fas, FasL, caspase-8 and -9, and the loss of mitochondrial membrane potential) was significantly triggered by magnolol (Figure 3 and Figure 6M–P). Protein levels of MCL-1, C-FLIP, and XIAP were effectively suppressed with magnolol treatment in CRC in vitro and in vivo (Figure 2F–I and Figure 6E–H). 

In conclusion, this study reveals that magnolol induces apoptosis through extrinsic/intrinsic pathways and inhibits NF-κB signaling through PKCδ inactivation in CRC. We suggested that the induction of apoptosis through extrinsic/intrinsic pathways and the suppression of PKCδ/NF-κB signaling are associated with magnolol-inhibited tumor progression in CRC in vitro and in vivo.

## 4. Materials and Methods

### 4.1. Reagents

Briefly, 3-(4, 5-Dimethylthiazol-2-yl)-2,5-diphenyltetrazolium bromide (MTT), magnolol and dimethyl sulfoxide (DMSO) were all purchased from Sigma-Aldrich (St.Louis, MO, USA). In addition, NF-κB inhibitor 4-N-[2-(4-phenoxypheny) ethyl]quinazoline-4,6-diamine (QNZ), AKT inhibitor LY294002, ERK inhibitor PD98059, P38 inhibitor SB203580 and JNK inhibitor SP600125 were all purchased from Selleckchem (Houston, TX, USA).

### 4.2. Cell Culture

CT26 was purchased from Bioresource Collection and Research Center (BCRC), Hsinchu city, Taiwan. HT29 was kindly provided from Professor Jing-Gung Chung, China Medical University. Both cells were maintained in RPMI 1640 medium (Thermo Fisher Scientific, Fremont, CA, USA) with supplementary 10% fetal bovine serum (FBS), and 1% L-glutamine (2 mM), penicillin (100 U/mL), and streptomycin (100 mg/mL) (Gibco/Life Technologies, Carlsbad, CA, USA). Cells were incubated at 37 °C humidified incubator with an atmosphere containing 5% CO_2_.

### 4.3. Cell Viability (MTT Assay)

CT26 and HT29 cells were seeded in 96 wells (5000 cells/well) overnight and treated with 0, 25, 50, 75, 100 μM magnolol for 24 h. Medium was then replaced by 100 μL MTT buffer (1:9 = medium: MTT 5 mg/mL) for another 4 h. Next, MTT medium was refreshed by 100 μL DMSO and genteelly shake for 10 s. Finally, the signal from living cells was detected by SpectraMax iD3 microplate reader (Molecular Devices, San Jose, CA, USA). 

### 4.4. Transfection and Stable Clone Selection

Stable clones of CT26/*NF-κB-luc2* cells were established by using jetPEI™ reagent (Polyplus Transfection, Sélestat, France). Plasmid NF-κB-luciferrase2 plasmid (pGL4.32[luc2P/NF-kB-RE/Hygro]) (cat. No. E8491) was purchased from Promega (Madison, WI, USA). Detail procedure was described in previous study [46,47]. Hygromycin B (200 μg/mL) was used to selected cells expressed with NF-κB-luc2 (Santa Cruz Biotechnology, CA, USA). Luc protein expression signal was validated by IVIS 200 Imaging System (Xenogen, Alameda, CA, USA). 

### 4.5. Reporter Gene Assay

CT26/*NF-κB-luc2* cells (5000 cells/well) were seeded in a 96-well plate overnight, followed with 0–100 μM magnolol, 0–20 nM Indolactam V, 0–4 μM Rottlerin, or a combination treatment for 24 h. After treatment, 500 μM D-luciferin was added to 100 μL PBS 5 min before an IVIS 200 system 1 min scan. The NF-κB activity in CT26/*NF-κB-luc2* cells were then measured and quantified by IVIS 200 Imaging System and Living Image software (Version 2.20, Xenogen, Alameda, CA, USA), respectively, at unit photons/s/cm^2^/sr. Additionally, the relative intensity of NF-κB was also normalized by cell viability [48].

### 4.6. Caspase-3, -8, -9 Activity Analyses

CT26 and HT29 cells were seeded in a 12-well plate (2 × 10^5^ cells/well) overnight and treated with 0, 75, or 100 μM magnolol for 24 h. Cells were then collected by 2000 rpm centrifugation and stained with 100 μL FACS buffer (0.5% FBS in phosphate-buffered saline) containing 1 μL cleaved caspase-3 (CaspGLOW™ Fluorescein Active Caspase-3 Staining Kit, BioVision), cleaved caspase-8 (CaspGLOW™ Red Active Caspase-8 Staining Kit, BioVision) or cleaved caspase-9 (CaspGLOW™ Fluorescein Active Caspase-9 Staining Kit, BioVision) antibodies for 30 min in dark at 37 °C incubator. Expression pattern of above molecules was detected and quantified by NovoCyte flow cytometry and NovoExpress^®^ software (Agilent Technologies Inc., Santa Clara, CA, USA), respectively. All experiments were performed in triplicate and were repeated at least three times.

### 4.7. Fas and Fas-L Analysis

CT26 and HT29 cells were seeded in a 12-well plate (2 × 10^5^ cells/well) overnight and treated with 0, 75, or 100 μM magnolol for 24 h. Cells were then collected by 2000 rpm centrifugation and stained with 100 μL FACS buffer containing 1 μL FAS-FITC and FASL-PE antibodies for 30 min in dark (Thermo Fisher Scientific). The expression patterns of FAS and FAS-L were detected and quantified by NovoCyte flow cytometry and NovoExpress^®^ software, respectively. All experiments were performed in triplicate and were repeated at least three times.

### 4.8. Mitochondrial Membrane Potential (MMP) and Cellular Ca^2+^ Analysis

CT26 and HT29 cells were seeded in a 12-well plate (2 × 10^5^ cells/well) overnight and treated with 0, 75, or 100 μM magnolol for 24 h. Cells were then collected by 2000 rpm centrifugation and stained with 500 μL FACS buffer containing 4 μM 3,3′-dihexyloxacarbocyanine iodide (DiOC_6_) or Fluo-3/AM (2.5 μg/mL) dye for 30 min in dark (Thermo Fisher Scientific).

### 4.9. Invasion Tranwell Assay

Transwell chambers purchased from BD Biosciences (Franklin Lakes, NJ, USA) were coated with 50 μL matrigel one day before invasion assay. CT26 and HT29 cells were seeded in 10-cm plates (3 × 10^6^ cells/well) overnight and treated with 0, 75, or 100 μM magnolol, 4 μM Rottlerin or 0.5 μM QNZ for 24 h. After treatment, cell viability was rapidly determined with trypan blue, before 2 × 10^5^ viable cells were re-suspended in 300 μL serum-free medium and added to the upper chamber. The lower chamber was filled with 700 μL RPMI-1640 medium containing 10% FBS. After 48 h of migration, cells that invaded transwell membranes were fixed by 4% formaldehyde, stained by 0.5% crystal violet, photographed by microscope (Nikon ECLIPSE Ti-U, Minato City, Tokyo, Japan), and quantified by ImageJ software version 1.50 (National Institutes of Health, Bethesda, MD, USA) [49].

### 4.10. Western Blot

CT26 and HT29 cells were seeded in 10-cm plates (3 × 10^6^ cells/well) overnight and treated with 0, 75, or 100 μM magnolol for 24 h. In addition, for PKC activator evaluation, CT26 and HT29 cells were treated with 20 nM Indolactam V for 0.5, 2, or 4 h. Treated cells were collected and lysed by NP-40 lysis buffer containing both 1% protease inhibitor and 1% phosphatase inhibitor mixture II (Sigma-Aldrich). The protein concentration was measured by BCA Protein Assay Kit (Thermo Fisher Scientific). Forty micrograms of protein per group were separated by 6–12% SDS-page and transferred onto polyvinylidene difluoride (PVDF) membranes (EMD Millipore, Bedford, MA, USA) [50]. Immunoreactive proteins were probed and detected by Immobilon Western Chemiluminescent HRP Substrate (Pierce, Rockford, IL, USA) and ChemiDoc-It imaging system (UVP, Upland, CA, USA), respectively. All the protein expression levels were normalized by actin or phospho-protein/total-protein before comparing with the 0 μM magnolol group. 

### 4.11. Animal Experiment

Animal experiments were approved by Institutional Animal Care and Use Committee in China Medical University and followed by the guidelines for the use of laboratory animals. Six-week-old BALB/c (n = 12 for each experiment, repeated twice) and Cg-Foxn1^nu^/CrlNarl (NUDE) male mice (n = 15 for each experiment, repeated twice) were purchased from National Laboratory Animal Center, Taipei, Taiwan. Ten million CT26 and HT29 cells were subcutaneous injected into mice right flank for growing as tumor (Figure 5A). Mice were randomly separated into 3 groups after average tumor volume reached 100 mm^3^, included 0 mg/kg magnolol, 50 mg/kg magnolol and 100 mg/kg magnolol. Tumor volumes were recorded by digital caliper every four days and calculated by following formula: tumor volume = length × width^2^ × 0.523. Mice body weight was also recorded every four days (from day 0 to 20). Mice were sacrificed on day 20 and tumors were finally extracted, photographed, measured by digital scales and sliced for immunohistochemistry (IHC) staining. 

### 4.12. Mice Computer Tomography (CT)

CT26 and HT29 cells bearing animal were performed with whole-body CT scan on day 0 and day 20. After 1–3% isoflurane anesthetized, mice were arranged for computer tomography (NanoSPECT/CT^®^PLUS, Mediso Ltd., Budapest, Hungary) scanning. The following operation information of CT scan was listed [46]: Tube energy: 55 kVp × 145 μA; 360 projections; voxel size: 145 × 145 × 145 μM.

### 4.13. Hematoxylin and Eosin (H&E) Staining

Kidney, liver, and spleen extracted from mice on day 20 were fixed by 4% formaldehyde and embedded paraffin for further slicing. Slicing and H&E staining were performed by bio-check laboratories ltd (Taipei, Taiwan) as followed with regular procedure [51]. 

### 4.14. Immunohistochemistry (IHC) Staining

In brief, slices were dehydrated with serial decreasing percentage of ethanol and further procedure was followed by manufacturer IHC staining instructions (EMD Millipore, Burlington, MA, USA). Tumor tissue slices were stained by various primary antibodies, including P-PKC delta, P-ERK, P-AKT, P-NF-Κb p65, MMP-9, VEGF, CyclinD1, C-FLIP, MCL-1, cleaved-caspase-3, cleaved-caspase-8, and cleaved-caspase-9. Slides were imaged using a Nikon ECLIPSE Ti-U microscope under 100× magnification. Positive staining signals were finally quantified by ImageJ software [52]. All the protein expression levels were normalized by actin or phospho-protein/total-protein before comparing with the 0 mg/kg magnolol group.

### 4.15. Statistical Analysis

Results are presented as mean ± SD. The significant differences between control and magnolol treatments were analyzed by Student’s t-test and one-way ANOVA. *p* value < 0.05 and *p*-value < 0.01 were both defined as an indication of statistical significance.
